# Characterization of pathological remodeling in the chronic atrioventricular block cynomolgus monkey heart

**DOI:** 10.3389/fphar.2023.1055031

**Published:** 2023-01-19

**Authors:** Hiroko Izumi-Nakaseko, Kengo Sakamoto, Ai Goto, Ryuichi Kambayashi, Akio Matsumoto, Yoshinori Takei, Akira Takahara, Atsushi Sugiyama

**Affiliations:** ^1^ Department of Pharmacology, Faculty of Medicine, Toho University, Tokyo, Japan; ^2^ Ina Research Inc., Nagano, Japan; ^3^ Department of Aging Pharmacology, Faculty of Medicine, Toho University, Tokyo, Japan; ^4^ Department of Pharmacology and Therapeutics, Faculty of Pharmaceutical Sciences, Toho University, Chiba, Japan

**Keywords:** cynomolgus monkey, torsade de pointes, chronic atrioventricular block, pathological remodeling, *in vivo* proarrhythmia model

## Abstract

We studied time course of pathological remodeling occurring in the cynomolgus monkey hearts against persistent atrioventricular block condition (*n* = 10). The atrioventricular block induced the ventricular and atrial dilation followed by the ventricular hypertrophy. Interstitial fibrosis in the ventricle was also observed along with gradual increases in the plasma angiotensin II and aldosterone concentrations. These adaptations were associated with the changes in gene expression profiling reflecting fibrosis and hypertrophy. Atrioventricular block reduced the ventricular rate and cardiac output, but the ejection fraction and stroke volume increased, whereas the cardiac output was gradually restored to its basal level. Systolic/diastolic blood pressure after the atrioventricular block was kept equal to or lower than that before the block, according with lack of increase in the plasma catecholamine levels. Chronic atrioventricular block gradually prolonged the QRS width and JT interval, leading to the QT interval prolongation in conscious state. 10 mg/kg of *dl*-sotalol hydrochloride induced torsade de pointes (TdP) in 6 out of 10 animals by 15 months. Animals showing longer QTcF under anesthesia after the atrioventricular block developed *dl*-sotalol-induced TdP earlier. No marked difference was observed in pharmacokinetics of *dl*-sotalol between 1 and 7 months after the atrioventricular block. Each TdP spontaneously terminated, reflecting a monkey’s relatively small “effective size of the heart (=∛(left ventricular weight)/wavelength of reentry)”. These fundamental knowledge will help better utilize the chronic atrioventricular block monkeys as an *in vivo* proarrhythmia model for detecting drug-induced TdP.

## Introduction

The chronic atrioventricular block canine model (the canine model) has been used for assessing proarrhythmic effects of plenty of drugs for >20 years, which is now considered to be one of the most established and reliable *in vivo* models of torsade de pointes (TdP) (Sugiyama, 2008; Loen et al., 2022). Previous studies have shown that the chronic atrioventricular block canine model concurrently possesses morphological, electrophysiological and pathological remodeling; including macroscopic and microscopic myocardial hypertrophy and increase of collagen fiber and extracellular space (Newman, 1978; Volders et al., 1998; Vos et al., 1998; Sugiyama et al., 2002a), downregulation of I_Ks_ and I_Kr_ (Volders et al., 1999; Takahara et al., 2011), and elevation of sympathetic drive, plasma atrial natriuretic peptide (ANP) and angiotensin II (Nishimura et al., 1990; Sugiyama et al., 2002b; Takahara et al., 2007). On the other hand, *in vivo* safety pharmacological analysis of new molecular entities is performed using monkeys, minipig and/or dogs according to ICH S7B Guideline (ICH Harmonised Tripartite Guideline, 2005). In our previous study using the chronic atrioventricular block model of minipig, *dl*-sotalol administration failed to induce torsade de pointes but led to the ventricular pauses followed by runs of multiple ectopic beats (Goto et al., 2019b), which has increased the need for monkey model of TdP. In order to meet such demand, we developed a chronic atrioventricular block monkey model (the monkey model) and have pharmacologically studied the model (Goto et al., 2020a; Goto et al., 2020b; Goto et al., 2021; Goto et al., 2022).

As far as we have examined the monkey model, it can assess the magnitude of risk of drugs for inducing TdP with high sensitivity and specificity similarly to the canine model (Goto et al., 2020a; Goto et al., 2020b; Goto et al., 2021; Goto et al., 2022; Sugiyama, 2008). Importantly, the drug-induced TdP spontaneously terminated in the monkey model unlike in the canine model (Goto et al., 2020a; Goto et al., 2020b; Goto et al., 2021; Goto et al., 2022; Sugiyama, 2008). This enables the monkey model to perform experiments in a repeated-measures design, which could reduce the total number of animals. While it is true that the monkey model is more predictive than the canine model when the metabolites and/or its metabolic pathways are unique to primates (Goto et al., 2020a; Goto et al., 2021; Goto et al., 2022), it is still unknown how long it takes for the monkey heart to complete the pathological remodeling after the onset of atrioventricular block, what the success rate of the proarrhythmia model creation is, and/or which factors are important for the model to complete the remodeling process.

In this study, we sought to answer those questions of the monkey model by assessing anatomical, electrophysiological, neurohumoral and pharmacological indices along with gene expression profiling. For this purpose, we performed several examinations as summarized in Table 1. The sensitivity of the model for detecting drug-induced TdP was tested by oral *dl-*sotalol administration. We propose that these assessments would help clarify the advantage and limitation of the chronic atrioventricular block monkeys as a proarrhythmia model for detecting the drug-induced TdP.

**TABLE 1:** Experimental protocol.

Variables	−1 week	0 month		1–7	8	9	10	11	12	13	14	15
		AVN ablation										
		Pre	Post	Every month								
Assessment of *dl*-sotalol induced TdP with a Holter ECG	✓	−	−	✓	✓	✓	✓	−	✓	✓	−	✓
Blood pressure, Lead II ECG (conscious and anesthesia)	✓	−	−	✓	✓	−	✓	✓	−	−	−	✓
Echocardiography	−	✓	✓	✓	−	−	−	−	−	−	−	✓
Chest radiographs	✓	−	✓	✓	✓	−	−	−	−	−	−	✓
Neurohumoral factors/Blood biochemistry/Hematology	✓	−	−	✓	✓	✓	✓	✓	✓	−	−	✓
Plasma concentration of *dl*-sotalol	−	−	−	✓*	−	−	−	−	−	−	−	−
Histology	−	−	−	−	✓**	−	−	−	−	−	−	−
Gene expression	−	−	−	−	✓**	−	−	−	−	−	−	−

✓, performed; −, not performed. AVN, atrioventricular node; TdP, torsade de pointes; ECG, electrocardiogram. *The plasma concentrations of *dl*-sotalol were measured in all animals except for #3 (*n* = 9) at 1 month and in #2 and #8 animals at 7 months after the onset of atrioventricular block. The #2 and #8 ones had shown torsade de pointes attacks for ≥3 times by 7 months. **Intact animals (*n* = 4) and atrioventricular block ones (#2 and #8) at 8 months after the onset of atrioventricular block were examined.

## Materials and methods

All experiments were approved by the Committee for Research at Ina Research Inc. (Nagano, Japan) (No. INA2008022), and performed according to the Guidelines for Animal Experiments, Ina Research, Inc., and the Guiding Principles in the Use of Animals in Toxicology, which were adopted by the Society of Toxicology in 1989. The total number of 14 male Vietnamese cynomolgus monkeys, *Macaca fascicularis* aging 4–6 years were used, which were purchased from Nafovanny Joint Venture Company (Dong Nai, Vietnam). The animals were kept in individual cages on a 12 h light (7:00–19:00) to dark (19:00–7:00) cycle. The ventilation provided a total air exchange rate of 15–26 times per hour. The room temperature was maintained at 22.0–28.0°C, and relative humidity was 40–80%. The animals had access to water *ad libitum* and received 100 g of food pellets once a day.

### Experimental design

The 14 male cynomolgus monkeys were divided into intact group (*n* = 4) and atrioventricular block group (*n* = 10), since the success rate of the model creation was roughly estimated to be 65% based on our preliminary experiments. The experimental protocol conducted in this study was summarized in Table 1. Holter electrocardiogram was recorded in conscious state to assess whether *dl*-sotalol could induce TdP. Blood was sampled to measure the concentrations of neurohumoral factors, biochemical and hematological variables, and *dl*-sotalol. Echocardiogram and chest radiograph were taken under anesthesia. Electrocardiogram and blood pressure were measured both in conscious state and under anesthetic condition. Analysis of histology and gene expression profiling were performed at 8 months in two animals which had developed TdP most frequently by monthly *dl*-sotalol challenge, which was compared with those obtained from four intact animals.

### Production of complete atrioventricular block

The catheter ablation technique was employed according to the previous reports (Goto et al., 2020a; Goto et al., 2020b; Goto et al., 2021; Goto et al., 2022; Sugiyama, 2008). In brief, the animals (*n* = 10) were anesthetized with ketamine hydrochloride (5–20 mg/kg, i.m.) and xylazine (1–2 mg/kg, i.m.). Under spontaneous respiration, a clinically available 5-French quad-polar electrodes catheter (Cordis-Webster, Baldwin Park, CA, United States) was inserted through the right femoral vein under sterile condition and its tip was positioned across the tricuspid valve under the guide of bipolar electrogram from the distal electrode pair. After the optimal site for the atrioventricular node ablation was determined as previously described (Sugiyama et al., 2002b), the radiofrequency energy of 20 W was delivered for 10 s from the tip electrode to an indifferent patch electrode positioned on the animal’s back. The endpoint of this procedure was the development of the complete atrioventricular block with an onset of stable idioventricular escape rhythm. Proper care was taken for the animals including careful observation, and use of analgesics and antibiotics including penicillin and streptomycin until their general condition was recovered. When the atrioventricular conduction was spontaneously recovered before the pharmacological assessment, the catheter ablation was conducted again.

### Experimental protocol in conscious state

#### Blood pressure and electrocardiogram assessment

After the animal sat at the monkey chair in conscious state, the blood pressure at the left brachium was measured using a cuff system with digital electrical manometer (BP-88V, Colin Medical Technology Co., Aichi, Japan). The surface lead II electrocardiogram was obtained from the limb electrodes with a polygraph system at a paper speed of 50 mm/s for 10–15 s (model 363, GE Healthcare Japan, Tokyo, Japan), and 10 consecutive complexes were used for analysis.

#### *dl*-sotalol challenge

Holter electrocardiogram recorder (QR2100, Fukuda M-E Kogyo Co., Ltd. Tokyo, Japan) was set on the animal to obtain NASA (xiphoid process-manubrium) and CM5 (V5-manubrium) leads electrocardiogram for 24 h. Since in the canine model, each oral administration of 3 and 10 mg/kg of *dl*-sotalol was shown to induce TdP in 3 out of 4 animals (Goto et al., 2019a; Goto et al., 2019b), we adopted 10 mg/kg for testing the sensitivity of the monkey model for detecting drug-induced TdP. About 2 h after the start of electrocardiogram recording, a gelatin capsule containing 10 mg/kg of *dl-*sotalol hydrochloride was administered by oral gavage to assess the sensitivity of the animals for detecting the drug-induced TdP, which was performed before the atrioventricular block and repeated every month thereafter by 15 months except for 11 and 14 months (Table 1). Vehicle alone or moxifloxacin (100 mg/kg, p.o.) was administered to the animals which had developed TdP for ≥3 times by 8 months to confirm their specificity against torsadogenic events (Table 2), since 100 mg/kg of moxifloxacin was classified as intermediate risk and 10 mg/kg of *dl*-sotalol as high risk (Goto et al., 2020a; Goto et al., 2021; Whittaker et al., 2021). Twenty-four h after the start of electrocardiogram recording, Holter electrocardiogram recorder was removed from the animals, and electrocardiogram was examined by using the analyzing system (HS1000, Fukuda M-E Kogyo Co., Ltd.). TdP was defined as a polymorphic ventricular tachycardia associated with QT interval prolongation prior to its onset, consisting of six beats or more twisting QRS complexes around the baseline (Dessertenne, 1966). We counted single and multiple premature ventricular beats in addition to TdPs from 1 to 3 h (around T_max_) after the oral administration of *dl*-sotalol. Each arrhythmic severity was quantified using arrhythmic score based on a previous report by Smoczynska et al. (2019), which was the average of the three most severe arrhythmic events during the analysis period of 2 h as depicted in Table 3. The differences in the observation methods of arrhythmic events between ours and Smoczynska et al.’s (2019) were the state of animals (conscious instead of anesthetized), administration route (p.o. instead of i.v.), observation period (2 h instead of 10 min), timing (around T_max_ instead of after the start of infusion), and species (monkeys instead of dogs).

**TABLE 2:** Time courses of the onset of torsade de pointes (TdP) in the atrioventricular block (AVB) monkey after oral administration of *dl*-sotalol hydrochloride (10 mg/kg).

Animal no.	−1 week	Month (s) after the onset of AVB												
		1	2	3	4	5	6	7	8	9	10	12	13	15
#1	−	−	−	−	−	−	n/r	−	−	−	−	−	−	−
#2	−	+	+	+	+	−^Ve^	−^M^, +	n/r	*					
#3	−	n/a^a^	−	−	+	−	n/r	+	+	−^Ve,M^	n/r^c^	n/r^c^	n/r^c^	−
#4	−	−	−	n/a^a^	−	−	n/r	−	−	−	−	−	−	−
#5	−	−	−	−	−	−	n/r	−	−	−	−	−	−	−
#6	−	−	−	−	−	−	n/r	−	−	−	−	−	−	−
#7	−	−	−	−	−	−	n/r	+	−	−	−	−	−	−
#8	−	−	−	−	+	+	−^M^, +	−^Ve^	*					
#9	−	−	−	−	−	−	n/r	−	−	−	−	+	−	−
#10	−	−	+	−	−	n/a^b^	n/r	−	−	−	−	+	−	+

+, TdP induced by *dl*-sotalol; −, TdP not induced by *dl*-sotalol; n/a, not available; n/r, not recorded; Ve, vehicle; M, moxifloxacin in a dose of 100 mg/kg, p.o.; and *, autopsy.

^a^The animal was re-ablated because of the spontaneous recovery of the atrioventricular conduction.

^b^Electrocardiogram was not recorded due to the malfunction of Hoter recorder.

^c^ *dl*-Sotalol challenge was not performed in the animals having shown ≥3 times of TdP based on the 3Rs rule.

**TABLE 3:** Time courses of the arrhythmic severity in the atrioventricular block (AVB) monkey 1–3 h after oral administration of *dl*-sotalol hydrochloride (10 mg/kg).

Animal no.	−1 week	Month (s) after the onset of AVB												
		1	2	3	4	5	6	7	8	9	10	12	13	15
#1	.3	.3	.3	2.0	.3	.3		.7	.3	.3	.3	.3	.3	.3
#2	.3	13.0	26.3	32.7	30.7		44.0							
#3	.3		**5.0**	**5.0**	6.0	**5.0**		17.7	20.7					**5.0**
#4	.3	**3.0**	**5.0**		2.3	2.0		.3	.7	2.0	**3.7**	2.0	2.0	2.0
#5	.3	**5.0**	**5.0**	**4.0**	**5.0**	**5.0**		**5.0**	**4.3**	**5.0**	**5.0**	**5.0**	**5.0**	**5.0**
#6	.3	**3.0**	.3	2.3	2.7	2.0		2.7	2.0	**3.0**	**3.0**	**3.0**	**3.0**	**3.0**
#7	.3	2.3	3.7	2.0	**4.3**	2.0		10.3	**3.0**	2.0	**3.3**	**3.3**	**3.0**	**3.0**
#8	.3	**4.0**	2.3	**3.0**	30.7	6.0	30.7							
#9	.3	**5.0**	**4.3**	**5.0**	**3.0**	**5.0**		**5.0**	**3.0**	**5.0**	**5.0**	9.3	**5.0**	**5.0**
#10	.3	**4.0**	10.3	**5.0**	**5.0**			2.0	2.0	**5.0**	**5.0**	9.7	**5.0**	8.7

Arrhythmic events were counted 1–3 h after administration of *dl*-sotalol hydrochloride. Arrhythmic score was the average of the three most severe arrhythmic events during 2 h based on Smoczynska et al. (2019). Bold, ≥3 sets of run of 2–4 ectopic beats; gray shaded, TdP.

#### Neurohumoral, hematological and biochemical variables

Eleven mL of venous blood in total was withdrawn from the femoral vein of the monkey model in the morning before starting Holter electrocardiogram recording. Seven mL of the blood sample was added into disodium ethylenediaminetetraacetic acid (EDTA), which was centrifuged at 1,600 × *g* for 10 min to obtain its plasma. The plasma sample was used for measuring concentrations of neurohumoral factors; namely, aldosterone and angiotensin II with radioimmunoassay; adrenaline, noradrenaline and dopamine with high performance liquid chromatography; ANP with chemiluminescent enzyme immunoassay; brain natriuretic peptide (BNP) with chemiluminescent immunoassay; and plasma renin activity with enzyme immunoassay. The quantitative assay of angiotensin II was performed at SRL Medisearch Inc. (Tokyo, Japan), and that of the others were done by BML, INC. (Tokyo, Japan). One mL of the blood sample was added into EDTA, which was assayed with a hematological analysis system (ADVIA120, Siemens Healthineers, Forchheim, Germany) at Ina Research, Inc. (Supplementary Tables S2A, B). Three mL of the blood sample was added into heparin sodium and centrifuged at 1,600 × *g* for 10 min to obtain its plasma, which was assayed with biochemical analysis system (Automatic analyzer, model 7170, Hitachi High-Tech Corporation, Tokyo, Japan) at Ina Research, Inc. (Supplementary Tables S2A, B).

#### Plasma *dl*-sotalol concentration

To examine the possibility that changes in pharmacokinetic profile after the atrioventricular block may affect pharmacodynamic observation, the plasma concentration of *dl*-sotalol was measured in all animals at 1 month, and in the animals having shown ≥3 times of *dl*-sotalol-induced TdP attacks by 7 months. The blood was sampled from the cephalic vein at 1, 2, 4, and 8 h after the drug administration in conscious state. The plasma concentration of *dl-*sotalol was measured using a high-performance liquid chromatographic system at Ina Research Inc.

### Experimental protocol under anesthesia

The animals were anesthetized with ketamine hydrochloride (5–20 mg/kg, i.m.) and xylazine (1–2 mg/kg, i.m.) under spontaneous respiration. The dose level of xylazine in this study, of which standard one was reported to be .5 mg/kg, i.m. (Flecknell, 2016), may have a potential to enhance the decrease of the heart rate and blood pressure.

#### Echocardiogram

After the animals were placed in left lateral recumbency, echocardiogram was recorded using an ultrasound system (SSD-α7, Hitachi Aloka Medical, Ltd., Tokyo, Japan) with a probe (5.0 MHz, UST-5294–5, Hitachi Aloka Medical, Ltd.). A long axis and 4-chamber views were obtained from the left sternal border and the apex, respectively. Using the M-mode echocardiograms, left ventricular internal diameter (LVID) at end-diastole (LVIDd), left ventricular internal diameter at end-systole (LVIDs), left ventricular posterior wall thickness at end-diastole (LVPWd), interventricular septum thickness at end-diastole (IVSd), left atrial diameter (LAD), ejection fraction (EF), stroke volume (SV) and cardiac output (CO) were measured. The diameter of the inferior vena cava was measured using the M-mode echocardiograms from the subcostal margin. The LVIDd and LVIDs were used to estimate left ventricular volume with the Teichholz formula: left ventricular volume $=\frac{7}{\left(2.4+LVID\right)}\times {\left(LVID\right)}^{3}$ . The EF was calculated as $EF\;\left(\%\right)=\frac{SV}{left\;ventricular\;end-diastolic\;volume}\times 100$ , and SV was done as SV = (left ventricular end-diastolic volume)−(left ventricular end-systolic volume). Left ventricular wall mass (g) was estimated by [(IVSd + LVIDd + LVPWd)^3^–(LVIDd)^3^]×1.05 (Armstrong and Ryan, 2019).

#### Chest radiograph and electrocardiogram

Chest radiograph was taken with X-ray equipment (Sirius 125 MX, Hitachi Medical Corporation, Tokyo, Japan). Cardiothoracic ratio (%) was calculated as follows: maximal horizontal cardiac diameter/maximal horizontal thoracic diameter x 100. Normal cardiothoracic ratio in male cynomolgus monkey was reported to be 56–59% (Xie et al., 2014). The surface lead II electrocardiogram was obtained from the limb electrodes with a polygraph system in the same manner as that in conscious state, which can provide electrophysiological information of the monkey model in the absence of increased sympathetic tone as observed in conscious state. Bazett’s formula QTcB = QT/RR^0.5^ (Bazett, 1920) with RR interval given in seconds was used for conscious state, and Fridericia’s formula: QTcF = QT/RR^0.33^ (Fridericia, 1920) with RR interval given in seconds was applied for anesthetic condition based on the previous reports (Holzgrefe et al., 2014; Goto et al., 2022).

#### Histological analysis

The animals having developed TdP for ≥3 times by 7 months (*n* = 2, Table 2) were anesthetized with thiopental sodium (25 mg/kg, i.v.). After exsanguination, the heart was excised and plunged into saline. After the heart was rinsed with saline, the transmural specimen was obtained from the left ventricular free wall. The specimen was fixed in 10% neutrally buffered formalin, and embedded in paraffin. Paraffin sections in 4-μm thickness were stained with hematoxylin-eosin and Azan, which were examined microscopically. The hearts from the intact animals (*n* = 4) were assessed as control in the same manner.

#### Microarray analysis

The rest of the left ventricular free wall not used for histological studies (≥50 mg) was employed for the microarray analysis (*n* = 2, atrioventricular block heart; *n* = 4, intact heart). Total RNA was isolated from those samples using RNeasy Fibrous Tissue Mini Kit (QIAGEN, Valencia, CA, United States of America). Clear peaks of a marker, 18S rRNA and 28S rRNA with a modest elevation of the baseline, were confirmed in the electropherogram by Agilent 2100 Bioanalyzer (Agilent Technologies Japan, Ltd., Tokyo, Japan). The ratio of A260/A280 was in the range of 2.01–2.05. These data indicate low degradation and high purity of the total RNA samples. The gene expression profiling was performed by Bio Matrix Research, Inc. (Chiba, Japan). The protocol is summarized as following; 100 ng of total RNA was used to generate biotin-tagged cRNA using GeneChip^®^ 3′IVT Express Kit (Affymetrix, Santa Clara, CA, United States of America); the biotin-tagged cRNA was hybridized to GeneChip^®^ Rhesus Macaque Genome Array (Affymetrix); and the amount of bound ones was measured by staining and scanning fluorescence intensity according to Affymetrix protocols. The obtained data was analyzed by Affymetrix^®^ GeneChip^®^ Command Console^®^ Software and Affymetrix^®^ Expression Console™ with MAS5 algorithm (Affymetrix). The fold changes of mRNA related to cardiac fibrosis and hypertrophy, and those related to ion channels, pumps, an exchanger, connexins, Ca^2+^-handling proteins and receptors were calculated from the data of the atrioventricular block hearts and the intact ones using GeneSpring^®^ GX 10.0 (Agilent Technologies Japan, Ltd. Tokyo, Japan). Microarrays data are available by GSE199943, the GEO accession number.

### Drugs

The following drugs were purchased: ketamine hydrochloride (KETAMINE INJ. 5% FUJITA, Fujita Pharmaceutical Co., Ltd., Tokyo, Japan), xylazine (Seractal^®^ 2% injection solution, Bayer Yakuhin Ltd., Osaka, Japan), *dl-*sotalol hydrochloride (Sotacor^®^, Bristol-Myers Squibb Company, Tokyo, Japan), moxifloxacin (Avelox, Bayer Yakuhin, Ltd., Osaka, Japan) and thiopental sodium (Ravonal^®^ for injection, Mitsubishi Tanabe Pharma Co., Osaka, Japan).

### Statistical analysis

Data are expressed as mean ± s.e.m. Friedman test with Dunn’s multiple comparison test was used for repeated measured data in a group in Figures 2–5; Supplementary Tables S2A, B. Wilcoxon matched-pairs signed rank test was used for paired two groups in Figures 3, 4 (between before and 1 month after the production of atrioventricular block). Mann-Whitney *U* test was used for unpaired two groups (between TdP ≥3 and TdP 0–1) in Figures 2–4. A *p* value < .05 was considered to be statistically significant.

## Results

**FIGURE 1:**
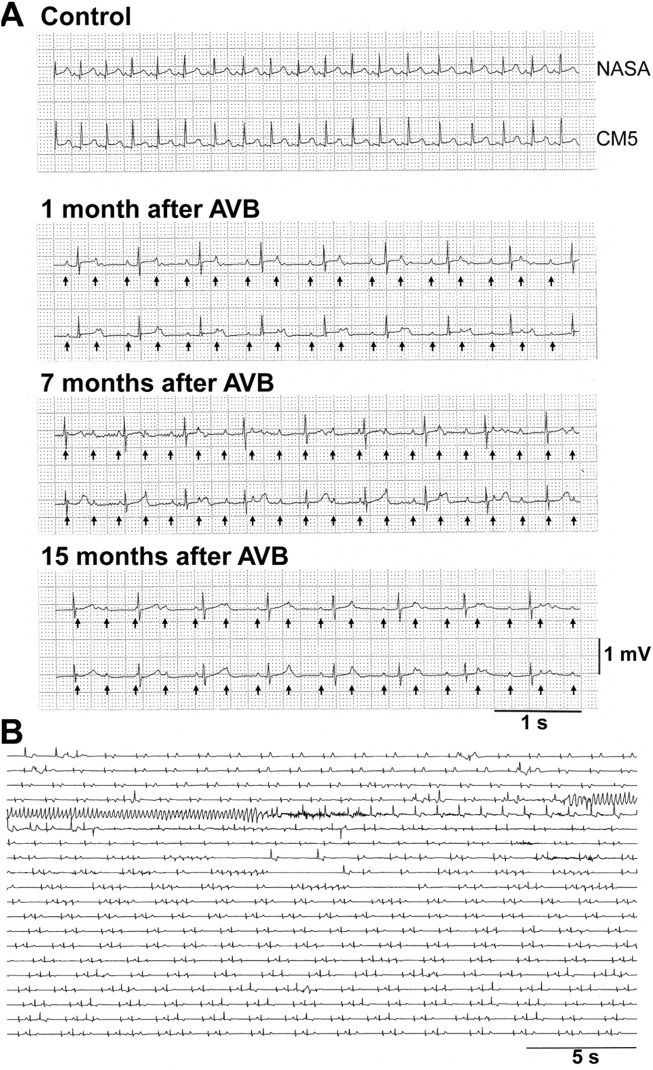
Representative traces of electrocardiogram in cynomolgus monkeys. **(A)** Representative traces by NASA (upper) and CM5 (lower) leads under non-ablated condition (Control) and 1, 7, and 15 months after atrioventricular node ablation (AVB) in the animal #10. Arrows indicate P-waves under complete atrioventricular block. **(B)** *dl*-Sotalol-induced torsade de pointes obtained by CM5 lead in the animal #2 at 6 months after the atrioventricular node ablation. The first arrhythmia episode was observed 2 h after the oral administration of 10 mg/kg of *dl*-sotalol hydrochloride. AVB, atrioventricular block.

The atrioventricular nodal ablation successfully eliminated the atrioventricular conduction in each animal. Since distress was not confirmed in any animal, we did not use analgesics. We did not observe any clinical sign of illness, either. As a humane endpoint, the signs of debility in the animals including cowering, sad expression and stop grooming were set to be criteria for considering euthanasia; however, no animal met these criteria. Typical electrocardiogram tracings before and after the atrioventricular nodal ablation are depicted in Figure 1. In two animals (#3 and #4), the atrioventricular conduction was spontaneously recovered by 1 and 3 months after the first intervention, respectively. We ablated their atrioventricular nodes again to eliminate the atrioventricular conduction as described in Table 2.

### Assessment of the onset of single and multiple premature ventricular beats, and TdPs

*dl-*Sotalol hydrochloride in a dose of 10 mg/kg was orally administered to the monkey model before and every month after the intervention as described in Table 1. By 2 months after the production of atrioventricular block, 8 out of 10 animals showed ventricular arrhythmias with ≥3 sets of run of 2–4 ectopic beats (arrhythmic score 3–5) following *dl*-sotalol administration as shown in Table 3, indicating that “the trigger” leading to the onset of TdP was developed in those animals during the initial 2 months. Consequently, 6 (#2, #3, #7, #8, #9, and #10) out of 10 animals developed TdP at least once during 15 months (Table 2). Typical trace showing the onset of *dl*-sotalol-induced TdP is depicted in Figure 1B. Four animals (#2, #3, #8, and #10) developed TdP for ≥3 times by *dl*-sotalol during the observation period, which was not induced by vehicle alone or by moxifloxacin (#2, #3, and #8) (Table 2). Each TdP observed in the four animals spontaneously terminated within 15 s (Supplementary Table S1).

### Echocardiography

**FIGURE 2:**
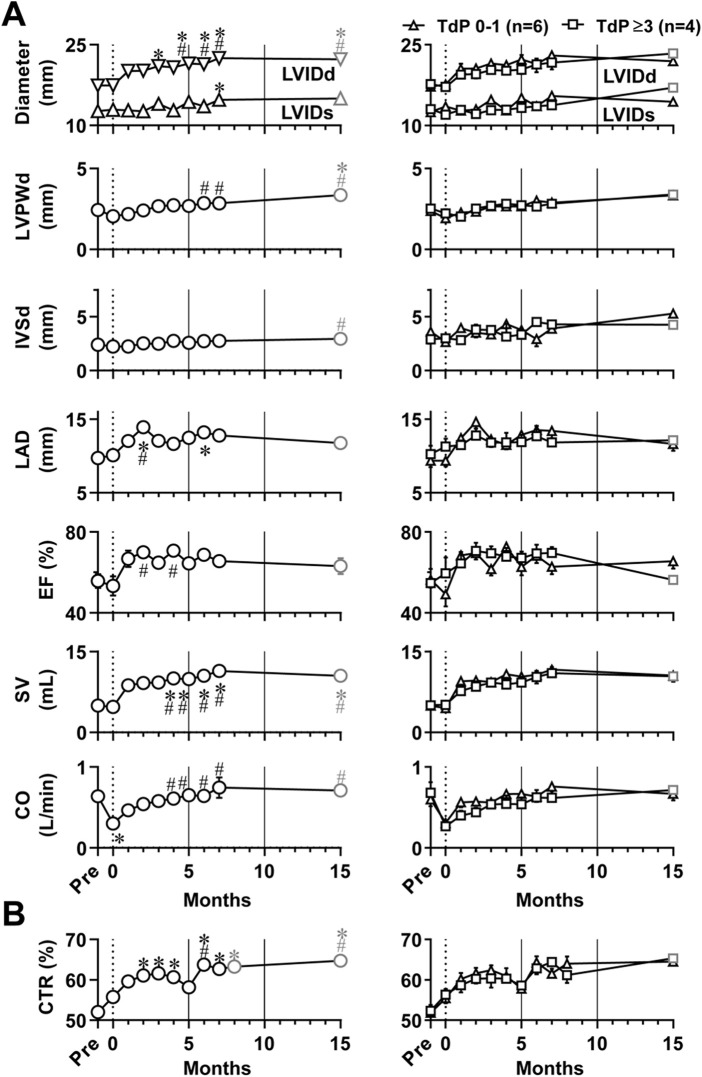
Time courses of changes in echocardiographic variables **(A)** and cardiothoracic ratio (CTR) of chest radiographs **(B)** in the atrioventricular block monkeys under anesthesia. The basal values (Pre) for the blood pressure, lead II electrocardiogram and chest radiographs were obtained 1 week before the production of atrioventricular block, whereas echocardiography was done just before the surgery for evaluation of Pre (Table 1). *p < .05 vs. Pre; ^#^ *p* < .05 vs. just after atrioventricular block (0 month) by Friedman test with Dunn’s multiple comparison test. In the left of each panel, mean values of all animals are shown (black symbols, *n* = 10 for Pre and 0–7 months; and gray symbols, *n* = 8 at 15 months); and in the right of it, averaged values in animals exerting torsade de pointes 0–1 time (TdP 0–1, triangles, *n* = 6) and those exhibiting torsade de pointes ≥3 times (TdP ≥3, black squares, *n* = 4 for Pre to 7 months; and gray squares, *n* = 2 at 15 months) by *dl*-sotalol challenge are depicted. Data are presented as mean ± s.e.m. LVIDd, left ventricular internal diameter at end-diastole; LVIDs, left ventricular internal diameter at end-systole; LVPWd, left ventricular posterior wall thickness at end-diastole; IVSd, interventricular septum thickness at end-diastole; LAD, left atrial diameter; EF, ejection fraction; SV, stroke volume; and CO, cardiac output.

Echocardiogram was recorded under the anesthesia as described in Table 1, and the time courses of changes in echocardiographic variables are summarized in Figure 2A. Basal control values before atrioventricular nodal ablation (Pre) for LVIDd, LVIDs, LVPWd, IVSd and LAD were 17.4 ± .4 mm, 12.7 ± .5 mm, 2.4 ± .1 mm, 2.4 ± .1 mm, and 9.7 ± .7 mm, whereas those for EF, SV and CO were 55.7 ± 3.4%, 4.98 ± .38 mL and .64 ± .06 L/min, respectively. When compared with those basal values before atrioventricular block (Pre), the atrioventricular block persistently increased LVIDd for 3–15 months except for 4 months and SV for 4–15 months; but sporadically increased LVIDs at 7 months, LVPWd at 15 months, and LAD at 2 and 6 months, whereas it transiently decreased CO at 0 month. When compared to the values immediately after the atrioventricular block (0 month), the atrioventricular block persistently increased LVIDd for 5–15 months, LVPWd for 6–15 months, SV and CO for 4–15 months; but sporadically increased LAD at 2 months, EF for 2 and 4 months, and IVSd at 15 months. Whether TdP was induced or not was determined regardless of the number of TdP occurred after the administration of *dl*-sotalol as summarized in Table 2. The ten animals were classified into two groups; namely, that exerting TdP 0–1 time (6 animals) and that exhibiting TdP ≥3 times (four animals) during the observation period (Figure 2A, right panels). No significant difference was detected in any of the echocardiographic variables between those two groups.

### Chest radiographs

Pleural effusion and pulmonary congestion were not observed during the observation period. The time course of cardiothoracic ratio calculated using chest radiographs is shown in Figure 2B. When compared with pre-operative basal values of 52.0 ± 1.2% before the production of atrioventricular block, the cardiothoracic ratio was 55.8 ± 1.5% (0 month) and increased for 2–15 months except that the increase was not significant at 5 months. No significant difference was detected in the cardiothoracic ratio between the groups of six animals exhibiting TdP 0–1 time and the group of four animals exhibiting TdP ≥3 times during the observation period (Figure 2B, right panel).

### Blood pressure and electrocardiographic variables in conscious state

**FIGURE 3:**
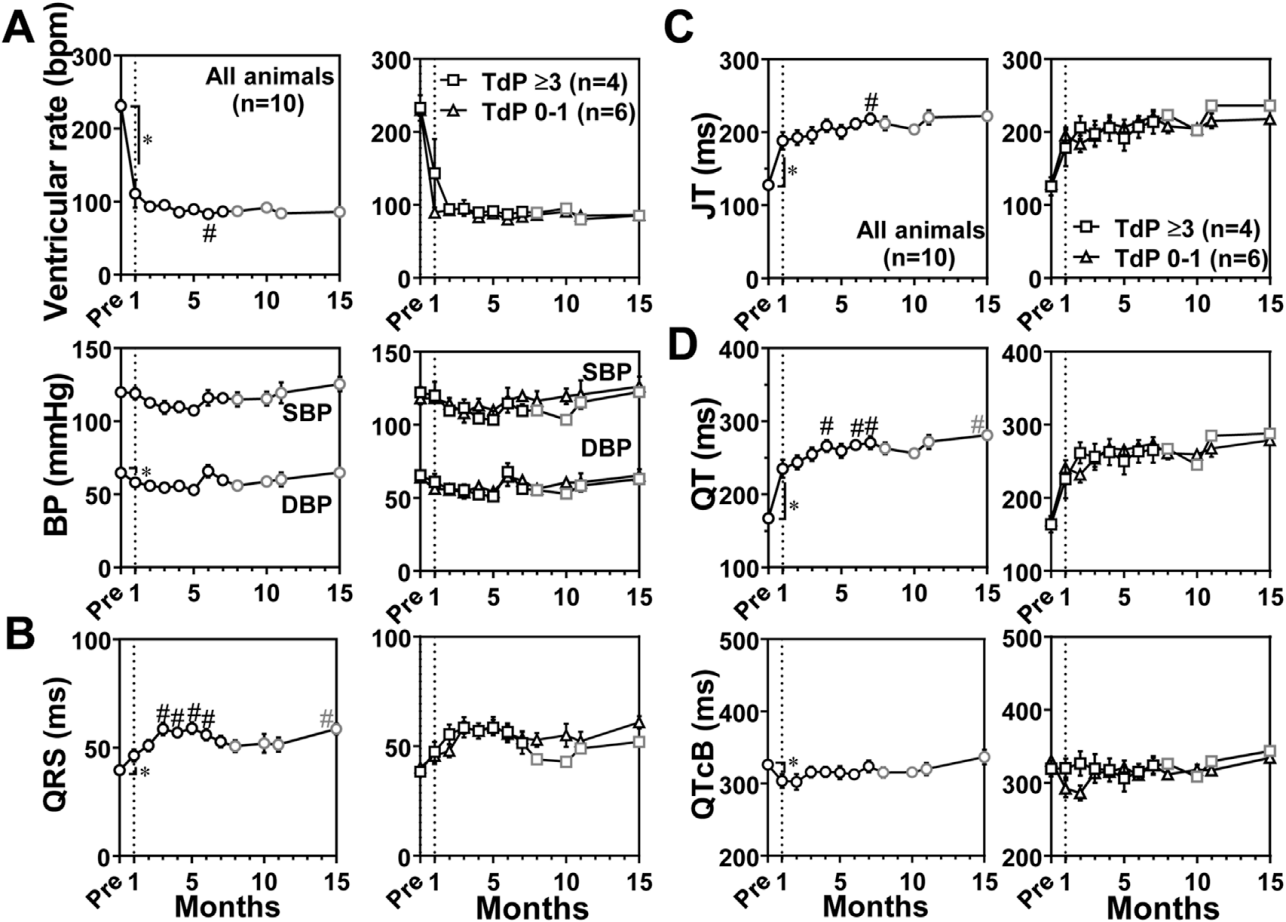
Time courses of changes in the ventricular rate and systolic/diastolic blood pressures (SBP/DBP) (BP) **(A)** and electrocardiographic variables including QRS width (QRS) **(B)**, JT interval (JT) **(C)**, and QT interval (QT) and QT interval corrected by Bazett’s formula (QTcB) **(D)** in the atrioventricular block monkeys in conscious state. In the left of each panel, mean values of all animals (All animals) are shown (black symbols, *n* = 10 for Pre and 0–7 months; and gray symbols, *n* = 8 for 8–15 months); and in the right of it, averaged values in animals exerting torsade de pointes 0–1 time (TdP 0–1, triangles, *n* = 6) and those exhibiting torsade de pointes ≥3 times (TdP ≥3, black squares, *n* = 4 for Pre to 7 months; and gray squares, *n* = 2 for 8–15 months) by *dl*-sotalol challenge are depicted. Data are presented as mean ± s.e.m. *p < .05, before atrioventricular block (Pre) vs. 1 month by Wilcoxon matched-pairs signed rank test; and #*p* < .05, vs. 1 month by Friedman test with Dunn’s multiple comparison test.

Blood pressure and electrocardiographic variables were recorded in conscious state as scheduled in Table 1, and the time courses of changes in blood pressure and electrocardiographic variables are summarized in Figure 3. Basal values before the production of atrioventricular block for the ventricular rate, systolic/diastolic blood pressure, QRS width, JT interval, QT interval, and QTcB were 231 ± 8 bpm, 120 ± 3/65 ± 1 mmHg, 40 ± 1 ms, 128 ± 6 ms, 167 ± 5 ms, and 326 ± 5 ms, respectively. The atrioventricular block decreased the ventricular rate and diastolic blood pressure to 111 ± 19 bpm and 58 ± 3 mmHg, respectively at 1 month, which was maintained up to 15 months (Figure 3A). It prolonged the QRS width, JT interval and QT interval to 46 ± 3 ms, 188 ± 12 ms, and 235 ± 12 ms, respectively at 1 month, which were further prolonged up to 15 months (Figures 3B–D). Meanwhile, QTcB was shortened to 304 ± 10 ms at 1 month. We performed subgroup analysis based on the number of TdP occurrence. No significant difference was observed in these electrocardiographic variables between the animals exerting TdP 0–1 time (*n* = 6) and TdP ≥3 times (*n* = 4) (Figure 3).

### Blood pressure and electrocardiographic variables under anesthetic condition

**FIGURE 4:**
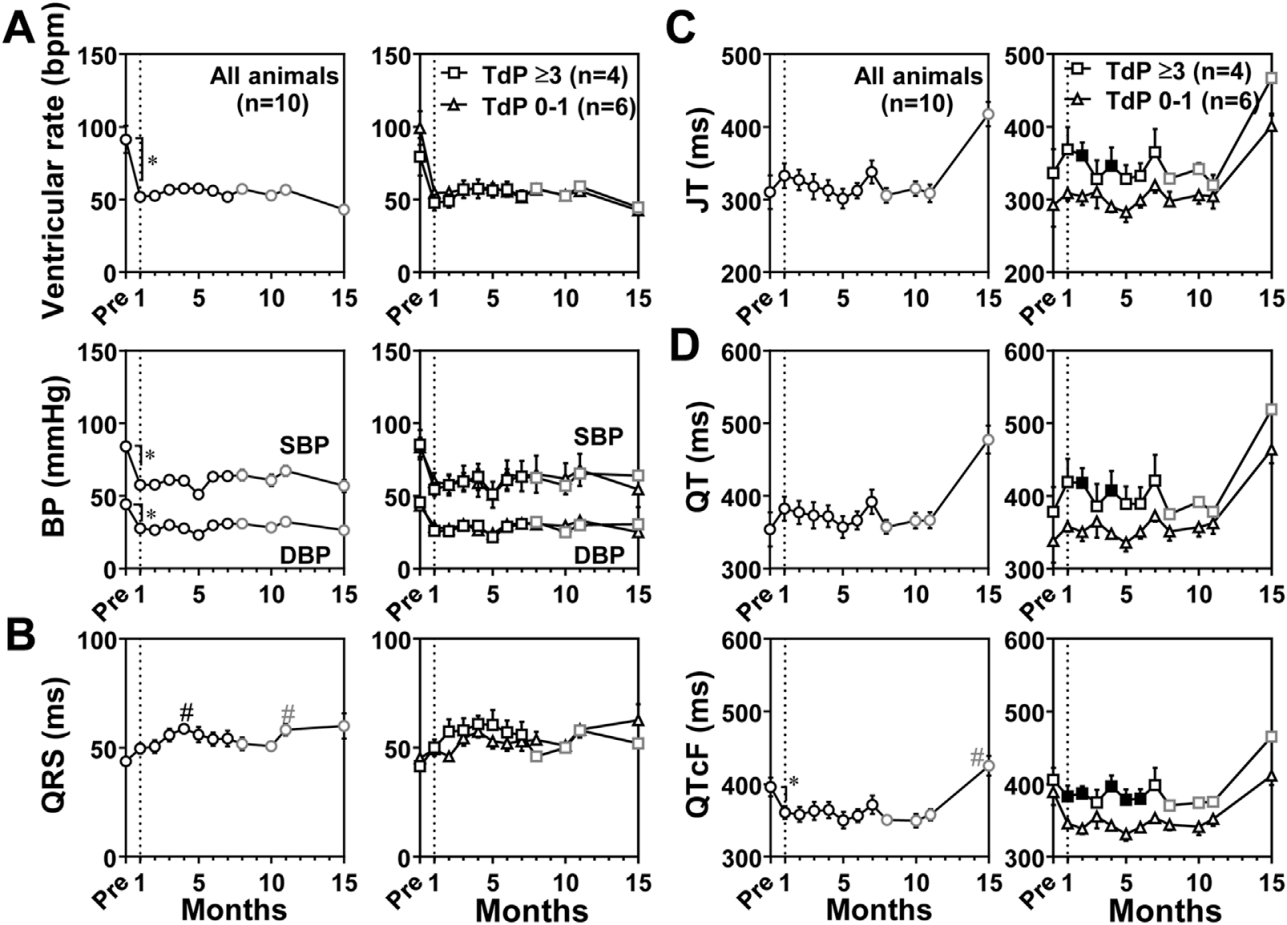
Time courses of changes in the ventricular rate and systolic/diastolic blood pressures (SBP/DBP) (BP) **(A)** and electrocardiographic variables including QRS width (QRS) **(B)**, JT interval (JT) **(C)**, and QT interval (QT) and QT interval corrected by Fridericia’s formula (QTcF) **(D)** under anesthesia. The diagram structure and definitions of abbreviations are the same as in Figure 3. Data are presented as mean ± s.e.m. *p < .05, before atrioventricular block (Pre) vs. 1 month by Wilcoxon matched-pairs signed rank test; and ^#^ *p* < .05, vs. 1 month by Friedman test with Dunn’s multiple comparison test. Filled symbols indicate *p* < .05 vs. the animals showing TdP 0–1 at the same month by Mann-Whitney *U* test.

Blood pressure and electrocardiographic variables were also recorded under anesthetic condition as scheduled in Table 1, and the time courses of changes in blood pressure and electrocardiographic variables are summarized in Figure 4. Basal values before the production of atrioventricular block for ventricular rate, systolic/diastolic blood pressure, QRS width, JT interval, QT interval and QTcF were 91 ± 9 bpm, 84 ± 3/44 ± 2 mmHg, 44 ± 1 ms, 310 ± 23 ms, 354 ± 23 ms, and 396 ± 13 ms, respectively. When compared with basal values before the production of atrioventricular block, the atrioventricular block significantly decreased the ventricular rate to 52 ± 3 bpm and the systolic/diastolic blood pressure to 58 ± 6/28 ± 3 mmHg at 1 month, which were maintained during the observation period. The atrioventricular block shortened the QTcF at 1 month (Figures 4B–D). When compared with values at 1 month, QRS width was prolonged at 4 and 11 months. QTcF were prolonged at 15 months. Compared with those in conscious state at 1 month, the anesthesia did not alter the QRS width, but decreased the ventricular rate by 59 ± 22 bpm, the systolic/diastolic blood pressure by 61 ± 5/30 ± 3 mmHg, and prolonged the JT interval by 144 ± 25 ms, the QT interval by 148 ± 25 ms. We performed the subgroup analysis as described above. The JT interval and QT interval at 2 and 4 months and QTcF for 1–6 months except that the prolongation was not significant at 3 months were prolonged in animals exerting TdP ≥3 times (*n* = 4) than in animals exerting TdP 0–1 time (*n* = 6) (Figures 4C, D).

### Neurohumoral profiles

**FIGURE 5:**
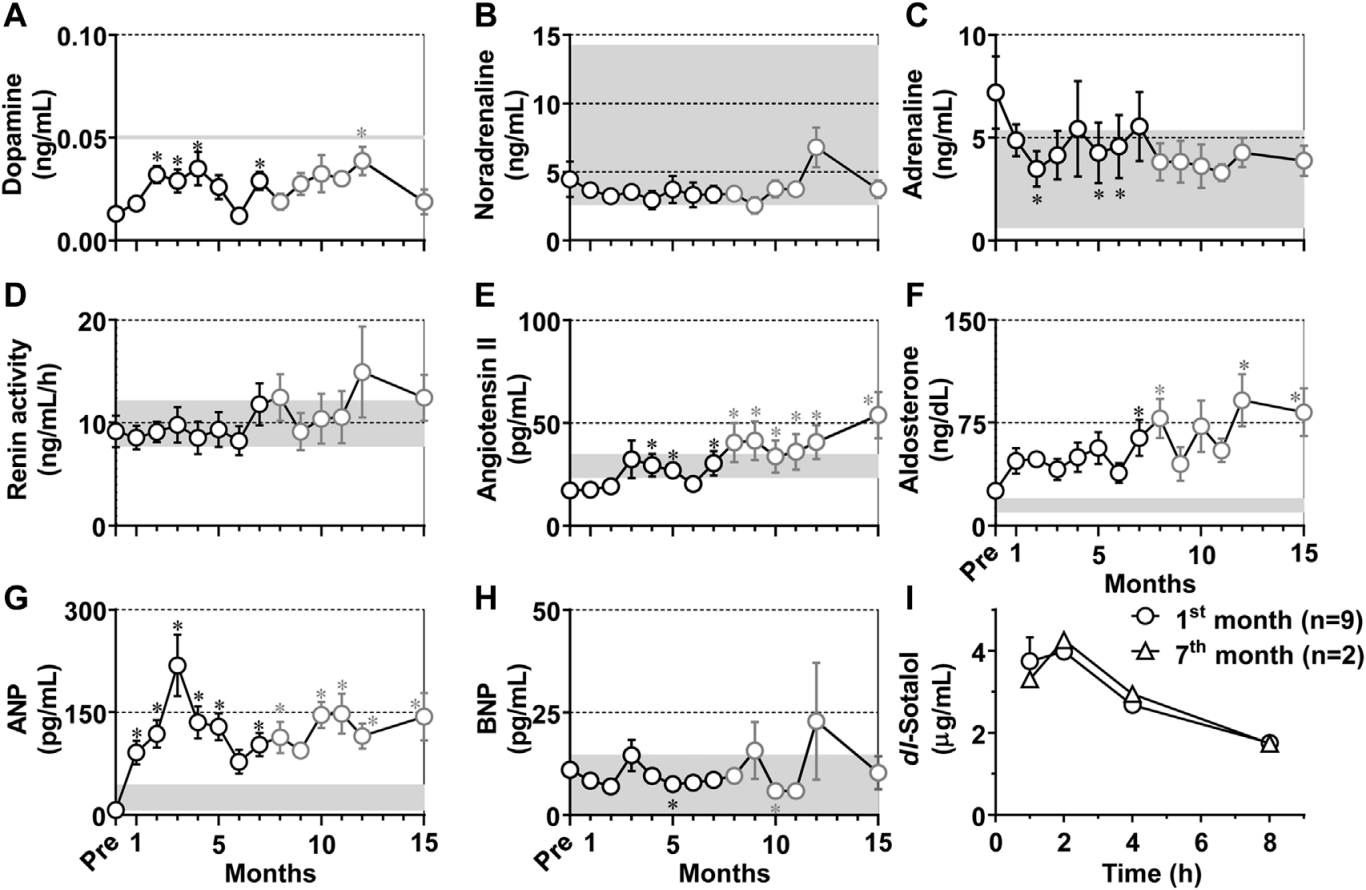
**(A–H)** Time courses in changes in the neurohumoral factors in the plasma of the atrioventricular block monkeys. *p < .05 vs. before atrioventricular block (Pre) by Friedman test with Dunn’s multiple comparison test. Data are presented as mean ± s.e.m. (*n* = 10 for Pre and 1–7 months, black symbols; and *n* = 8 for 8–15 months, gray symbols). Gray zones in all panels indicate physiological concentrations calculated by using data in previous reports (Mangan and Mason, 1958; Udelsman et al., 1987; Lenz T et al., 1991; Pai C et al., 2021). ANP, atrial natriuretic peptide; and BNP, brain natriuretic peptide. **(I)** Time courses of change in the plasma concentrations of *dl*-sotalol following oral administration of 10 mg/kg of *dl*-sotalol hydrochloride at 1 month after atrioventricular block (mean ± s.e.m., *n* = 9, circles), and at 7 months after atrioventricular block (mean, *n* = 2, triangles) in #2 and #8 animals which had developed torsade de pointes 4 and 3 times by *dl*-sotalol challenge, respectively (see Table 2).

Neurohumoral factors including dopamine, noradrenaline, adrenaline, renin activity, angiotensin II, aldosterone, ANP and BNP in plasma were measured in conscious state as scheduled in Table 1, and the time courses of changes in those neurohumoral variables are summarized in Figure 5. Their basal values before the production of atrioventricular block were .013 ± .002 ng/mL, 4.45 ± 1.30 ng/mL, 7.20 ± 1.76 ng/mL, 9.17 ± 1.53 ng/mL h, 17.1 ± 1.8 pg/mL, 25.3 ± 4.4 ng/dL, 7.23 ± .80 pg/mL, and 11.0 ± 1.3 pg/mL, respectively. The atrioventricular block increased dopamine for 2–4 months and at 7 and 12 months; lowered adrenaline at 2 months and for 5–6 months; elevated angiotensin II for 4–15 months except that the elevation was not significant at 6 months, aldosterone for 7–8 and 12–15 months, and ANP for 1–15 months except for 6 and 9 months; and decreased BNP at 5 and 10 months. Importantly, most of those fluctuations were within the physiological range except for ANP, angiotensin II and aldosterone. No significant change was observed in noradrenaline or renin activity.

### Pharmacokinetics of *dl-*sotalol

Plasma concentrations of *dl*-sotalol were measured at 1, 2, 4, and 8 h after the administration of *dl-*sotalol hydrochloride in all animals at 1 month except for #3 (*n* = 9) due to recovery of the atrioventricular conduction and in #2 and #8 animals at 7 months (*n* = 2), since the #2 and #8 animals had developed 4 and 3 times of TdP by 7 months, respectively (Table 2). The time courses of changes in the plasma concentration of *dl*-sotalol are summarized in Figure 5I. The mean C_max_, T_max_ and AUC_0–8h_ at 1 month were 4.74 μg/mL, 1.6 h, and 21 μg h/mL (*n* = 9), which were comparable to those measured at 7 months (4.26 μg/mL, 2 h, and 22 μg h/mL (*n* = 2), respectively).

### Hematology and biochemistry

Time course of the hematological and biochemical parameters is summarized in Supplementary Tables S2A, B. Several parameters showed alternations during the observation period; however, they were within the physiological range in cynomolgus monkeys (Schuurman and Smith, 2005; Park et al., 2016).

### Histology

**FIGURE 6:**
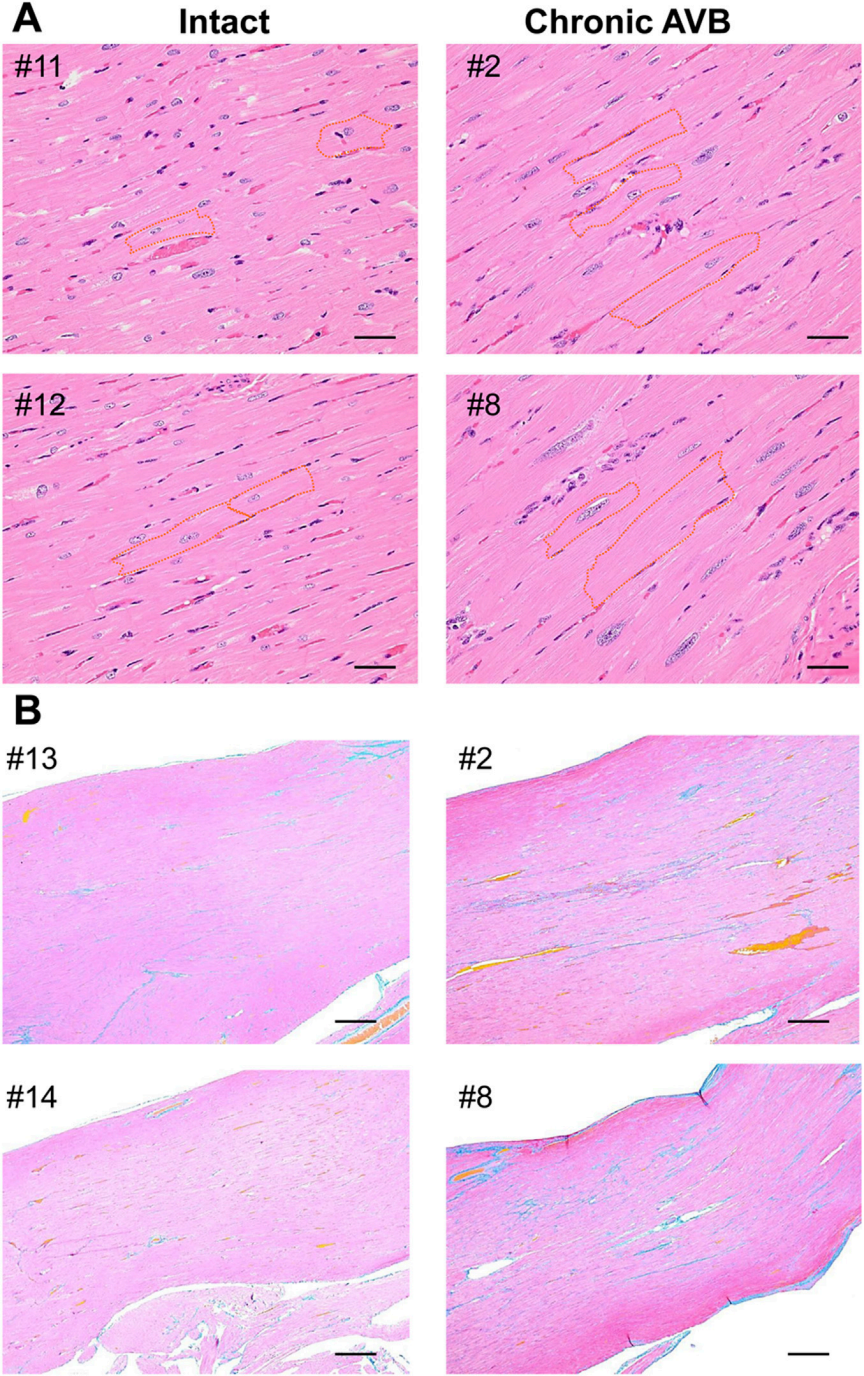
Microscopic photos of the free walls of the left ventricle obtained from intact animals (#11, #12, #13, #14, left) (Intact) and #2 and #8 animals at 8 months after the onset of atrioventricular block (Chronic AVB, right), which were stained with hematoxylin-eosin staining **(A)** and Azan **(B)**. Orange broken lines in panel A indicate outlines of cardiomyocytes. Scale bars: 20 μm **(A)** and 200 μm **(B)**.

At 8 months after the intervention, the histological preparations of the atrioventricular block hearts of #2 and #8 animals were examined in comparison with those from the intact animals (*n* = 4), and their typical photographs are shown in Figure 6. Longitudinal hypertrophy of cardiomyocytes and increase of interstitial fibrosis were observed in the chronic atrioventricular block hearts.

### Gene expression profile

Using the transmural tissue from endocardium to epicardium of left ventricle of the heart that was used for histological analysis, gene expression profile of ion channels, pumps, an exchanger, connexins, Ca^2+^-handling proteins and receptors was analyzed, and their results are summarized in Table 4. In the chronic atrioventricular block monkeys, gene expression levels of L-type calcium channel α_1D_ subunit (*CACNA1D*) and potassium inwardly-rectifying channel, subfamily J, member 2 (*KCNJ2*) increased among the cardiac ion channels; that of calcium/calmodulin-dependent protein kinase type 1D (*CAMK1D*) was elevated among Ca^2+^-handling proteins; and that of calcium-activated potassium channel beta four subunit (*KCNMB4*) belonging to the vascular ion channels was enhanced. No significant reduction in mRNA levels of variable cardiac K^+^ channel subunits was detected.

**TABLE 4:** Fold changes in expression levels of genes in the chronic atrioventricular block monkeys (*n* = 2) in comparison to intact ones (*n* = 4).

Category	Gene symbol	Fold change	Gene symbol	Fold change
Ion channels	*SCN5A*	.85	*KCNQ1*	.95
	*SCN7A*	1.21	*KCNJ2*	2.00*
	*SCN1B*	1.17	*KCNE1*	1.05
	*SCN2B*	1.39	*KCNMB4*	2.04*
	*KCNJ5*	.60	*CACNA1D*	3.39*
	*KCNJ8*	.59	*CACNA1C*	.99
	*KCNH2*	.88	*CACNB1*	1.85
Pumps, exchanger, connexins	*ATP2A2*	.91	*GJA1*	1.10
	*RYR2*	1.14	*GJC1*	.83
	*SLC8A1*	.73	*GJA5*	1.51
Ca^2+^-handling proteins	*CAMK1D*	2.06*	*PLN*	1.14
	*CAMK2A*	.67	*CALM3*	.77
	*CAMK2B*	1.27	*CASQ2* *^a^*	1.70
	*CAMK2D*	1.05		
Receptors	*ADRB2*	1.68	*ADRB1*	.65
Fibrosis^a^	*SERPINE2*	2.67*	*FN1*	2.52*
	*SERPINE2*	2.14*	*THBS1*	3.46*
	*SERPINE2*	2.25*		
Cardiac hypertrophy^a^	*TNFSF12/TWEAK*	2.44*	*TNFRSF12A*	3.20*
Fibrosis, hypertrophy, apoptosis^a^	*ANKRD1*	3.11*		
IFN-stimulated cytokine^a^	*IFI27*	.47*	*IFIT2*	.47*
Others^a^	*TMEM14C*	.39*	*RPL35A*	2.70*
	*TPT1/TPCT1*	.67	*SDF2*	.60
	*LPL*	.57	*USP14*	1.74
	*DEFB1/RHBD-1*	.65	*USP14*	1.73

^a^ Genes were selected from Supplementary Figure S1. * >2 or <.5 fold change vs. intact animals.

The raw gene expression levels between intact and chronic atrioventricular block monkey heart are shown in Supplementary Figure S1, indicating marked difference in some genes between the groups (Supplementary Figure S1, red points), which are listed in Table 4 (^#^). The histological findings in the chronic atrioventricular block monkey heart (Figure 6) were in accordance with increases in the expression levels of cardiac fibrosis-related genes, fibronectin 1(*FN1*) (Dobaczewski et al., 2010; Fan et al., 2012)*,* thrombospondin 1 (*THBS1*) (Rosini et al., 2018; Zhou et al., 2020) and serpin family E member 2 (*SERPINE2*) (Li et al., 2016); cardiac hypertrophy-related genes, TNF superfamily member 12 (*TNFSF12/TWEAK*) and TNF receptor superfamily member 12A (*TNFRSF12A/FN14*) (Méndez-Barbero et al., 2020); and a fibrosis-, hypertrophy- and apoptosis-related gene, ankyrin repeat domain 1 (*ANKRD1*) (Mikhailov and Torrado, 2008; Chen et al., 2014; Shen et al., 2015; Ling et al., 2017) (Supplementary Figure S1; Table 4). Meanwhile, the expression levels of IFN-stimulated cytokine genes; interferon alpha inducible protein 27 (*IFI27*) and interferon induced protein with tetratricopeptide repeats 2 (*IFIT2*) (Yang et al., 2017) decreased, indicating the absence of systemic inflammation at 8 months after the atrioventricular block. In addition, gene expression level of transmembrane protein 14C (*TMEM14C*) decreased, whereas that of ribosomal protein L35a (*RPL35A*) increased in the atrioventricular block heart.

## Discussion

We investigated the time course of pathological remodeling in the monkey hearts against atrioventricular block for the first time. *dl*-Sotalol induced ventricular arrhythmias by 2 months in 8 out of 10 animals (Table 3), indicating that “the trigger” leading to the onset of TdP was developed in their hearts within that period, which was in parallel with the time course of changes in the echocardiographic variables. However, contrary to our expectation based on previous knowledge from the canine model, in which the pathological remodeling completed within 4 weeks (Sugiyama 2008; Loen et al., 2022), the remodeling speed was much slower in the monkeys than in the dogs when the development of I_Kr_ inhibitor-induced TdP was used as a marker for completion of remodeling. Indeed, TdP was induced by *dl*-sotalol challenge in 6 out of 10 monkeys by 15 months after the atrioventricular block. Meanwhile, moxifloxacin or vehicle did not induce TdP in the animals (#2, #3 and #8), in which *dl*-sotalol did it, indicating that the monkey model may have enough sensitivity and specificity for assessing the torsadogenic potential of drug candidates.

### Morphological adaptation in the atrioventricular block monkey hearts

LVIDd, LAD, and cardiothoracic ratio increased shortly after atrioventricular block, followed by increases in LVIDs, LVPWd and IVSd (Figure 2). These observations indicate that the atrioventricular block can induce dilation of the ventricles and atria along with hypertrophy of the ventricular wall. In addition to these macroscopic changes, hypertrophy of the ventricular cardiomyocytes along with interstitial fibrosis was observed in the atrioventricular block hearts (Figure 6) of two animals having developed ≥3 times of TdP by 7 months after atrioventricular block (#2 and #8 in Table 2). Moreover, these histological findings could be partly supported by changes in the gene expression levels (Table 4).

### Functional responses in the cardiovascular system

Atrioventricular block reduced the ventricular rate (Figures 3, 4) and CO (Figure 2), but EF and SV increased, and the CO was gradually restored (Figure 2). Contrary to the canine models (Takahara et al., 2004; Takahara et al., 2007), systolic as well as diastolic blood pressure of the monkey model was equal to or lower than that before atrioventricular block (Pre) in conscious state (Figure 3) and under anesthesia (Figure 4). This observation could be partly explained by the 2-fold faster idioventricular automaticity rate in the monkey model than that in the canine one (Sugiyama et al., 2002a; Takahara et al., 2004; Loen et al., 2022), enabling the monkey heart to maintain the cardiac output within the physiological range, which may have hardly enhanced the sympathetic tone. Thus, the lack of increase in the afterload to the left ventricle might have slowed the atrioventricular block-induced pathological remodeling in the monkey heart.

### Neurohumoral responses

Holzgrefe et al. (2014) reported that the sinus rate in cynomolgus monkey in conscious state ranged from 77 to 250 bpm with telemetry system, indicating that the sinus rate of 231 ± 8 bpm in this study was relatively high. The plasma adrenaline level before the atrioventricular block was also high as shown in Figure 5, suggesting the presence of stress-induced increase of sympathetic tone. For example, the mounting of Holter electrocardiogram recorder without pharmacological sedation may have induced the stress to increase both of sinus and ventricular rates (Figure 3).

Atrioventricular block increased plasma dopamine level (Figure 5A), which was still within the physiological range of non-human primate (<50 pg/mL) (Udelsman et al., 1987). Meanwhile, atrioventricular block did not increase plasma noradrenaline or adrenaline level (Figures 5B, C), which was in the range of those reported for the intact monkeys that were 2.6–14.28 and .6–5.36 ng/mL, respectively (Mangan and Mason, 1958). Renin activity before the atrioventricular block was comparable to that in the intact monkeys; 9.1 ± 1.4 and 10.5 ± 1.7 ng/mL·h (Lenz et al., 1991) (Figure 5D), which tended to increase without attaining statistical significance after the atrioventricular block. These neurohumoral responses indicate that sympathetic tone may be hardly altered by the atrioventricular block, which was supported by the decrease of the blood pressure in conscious state (Figure 3) as well as under anesthesia (Figure 4). Angiotensin II was gradually elevated (Figure 5E), which exceeded the value of intact monkeys (29 ± 5.7 pg/mL) (Lenz et al., 1991) from 8 months. Plasma aldosterone concentration gradually increased (Figure 5F), and reached higher level than normal range of monkeys (10–20 ng/dL) (Lenz et al., 1991). Thus, increases in angiotensin II and aldosterone may partly contribute to progression of hypertrophy and fibrosis in the atrioventricular block heart (Bauersachs and López-Andrés, 2022; Watkins et al., 2012; Weber et al., 2013), increasing TdP susceptibility. ANP level increased for 1–15 months (Figure 5G), which was greater than that in intact cynomolgus monkeys (25.63 ± 18.62 pg/mL) (Pai et al., 2021) and comparable to that in the canine model (88 ± 18 pg/mL) (Takahara et al., 2007), indicating the presence of stretch of the atria of the atrioventricular block heart, which was in accordance with an increase of LAD (Figure 2A). Meanwhile, BNP level was not elevated and was much lower than that of the canine model (43 ± 10 pg/mL) (Takahara et al., 2007), suggesting lack of significant stretch of the ventricle after the atrioventricular block, which can be partly brought by relatively faster idioventricular rate and lower blood pressure compared with those in the canine model. The ANP level transiently returned to the basal level at 6 months, and gradually increased during the experimental period. The secretion of ANP is generally increased by stretch of atrial wall, which could be estimated by LAD. It should be noted that the LAD peaked at 2 months in this study, which preceded the peak of ANP by 1 month (Figure 2). Also, angiotensin II and aldosterone levels showed a similar time course to ANP. These findings indicate that compensatory neurohumoral responses may accelerate at 6 months after atrioventricular block in the monkey model.

### Electrocardiographic changes in the atrioventricular block monkey hearts

In conscious state, the QRS width was mildly prolonged after the onset of atrioventricular block (Figures 3B, D) possibly due to the pacemaker shift from sinus node to idioventricular automaticity derived from Purkinje fibers (Sugiyama et al., 2002b), which would have altered the order of ventricular depolarization as well as repolarization, secondarily prolonging the QT interval. The QRS width prolonged for 3–6 months and at 10 months and JT interval was prolonged at 7 months compared to 1 month (Figure 3). Prolonged QRS width reflected the increased fibrosis among the cardiomyocytes as well as the enlarged and hypertrophied heart. Mild and gradual prolongation of JT interval can be partly associated with upregulation of *CACNA1D* mRNA level (Table 4), suggesting that an inward cation current might be increased in the *in vivo* heart as discussed below. Thus, the QT interval prolongation consisted of lengthening of the QRS width and JT interval under the lower ventricular rates in conscious state (Figure 3).

*CACNA1D* codes low voltage-activated L-type Ca^2+^ channel Ca_V_1.3, which has been reported to be expressed in the supraventricular tissue of the normal adult heart, including the atria, sinoatrial and atrioventricular nodes (Mangoni et al., 2003; Baig et al., 2011; Zhang et al., 2011). Since Ca_V_1.3 has been reported to be expressed in the left ventricles of failing human heart (Srivastava et al., 2020), increase in gene expression level of *CACNA1D* in this study might reflect the pathological remodeling in the atrioventricular block heart. Moreover, Ca_V_1.3 has been reported to show slow voltage-dependent inactivation process (Platzer et al., 2000), increase of which could have partly contributed to the prolongation of JT interval (Figure 3).

Under anesthetic condition, the JT interval, QT interval and QTcF was longer in the animals exerting larger number of TdP (Figure 4), which was not observed in conscious state (Figure 3). These findings indicate that the chronic atrioventricular block could have more greatly reduced the magnitude of repolarization reserve in those animals, which might explain the delay in ventricular repolarization under anesthesia, since ketamine has been reported to inhibit I_Kr_, I_K1_ and I_to_ in cardiomyocytes (Endou et al., 1992; Zhang et al., 2013). It should be noted that QTcB and QTcF were shortened at 1 month compared with basal values before the production of atrioventricular block (Figures 3D, 4D). The gene expression level of *KCNJ2,* an inwardly-rectifying potassium channels Kir2.1, increased when measured at 8 months (Table 4), which might be at least in part associated with the shortening of corrected QT intervals at 1 month. On the contrary, no significant reduction in mRNA levels of various cardiac K^+^ channel subunits was observed by 8 months (Table 4) unlike in the canine models (Takahara et al., 2011), which might partly explain why it takes so long to complete the model. Along with cardiohemodynamic and neurohumoral responses, these observations suggest that additional manipulation to increase the ventricular load (use of midodrine or droxidopa to increase the afterload), and/or to reduce the ventricular rate (use of ivabradine or zatebadine to suppress HCN channels) would shorten the time to complete the model and improve the success rate. While cardiac remodeling could be completed by 1 month to induce drug-induced TdP after the production of atrioventricular block in most of the dogs (Sugiyama 2008), the repolarization reserve was diminished in only 4 out of 10 cynomolgus monkeys by 1 month (Figures 4C, D). Thus, those may partly depend on the difference in the blood pressure and ventricular rate between dogs and monkeys after the atrioventricular block.

### Potential mechanisms for spontaneous termination of TdP

In the chronic atrioventricular block monkey heart, each of the *dl*-sotalol-induced TdP spontaneously terminated, which is consistent with that reported for the acute atrioventricular block rabbit model (Hagiwara et al., 2017); however, in the chronic atrioventricular block canine model, most of TdP degenerated into ventricular fibrillation (Sugiyama, 2008; Smoczynska et al., 2019). In order to better understand underlying mechanism for spontaneous termination of TdP, we calculated “effective size of the heart (*I*)” for spiral rotation (Panfilov, 2006) using the formula: *I* = $\frac{\sqrt[{3}]{left\;ventriclar\;wall\;mass}}{period\;of\;spiral\;wave\;rotation}$ . The number of turns of a spiral in a given tissue depends on the ratio of tissue size to wavelength of a reentrant wave (Panfilov, 1999; Panfilov, 2006). The left ventricular wall mass was calculated using IVSd, LVPWTd and LVIDd of the echocardiography, which was 11.4 g for the chronic atrioventricular block monkey heart at 7 months (Figure 2), 75.4 g for the chronic atrioventricular block canine heart (Takahara et al., 2007), 179.3 g for the normal human adult heart (Feigenbaum, 1986) and 3.6 g for the intact male New Zealand white rabbit heart (Fontes-Sousa et al., 2006). Meanwhile, the frequency of TdP wave for those was estimated to be 2–5 Hz for the chronic atrioventricular block monkey heart (Goto et al., 2020a; Goto et al., 2020b; Goto et al., 2021), 5–6 Hz for the chronic atrioventricular block canine heart (Izumi-Nakaseko et al., 2017; Goto et al., 2018; Goto et al., 2019a; Goto et al., 2019b), 3–4.5 Hz for the human adult heart (Rosso et al., 2021) and 3–6 Hz for the acute atrioventricular block rabbit heart (Hagiwara et al., 2017), respectively. As a results, *I* was calculated to be 4.5–11.2 for the chronic atrioventricular block monkey heart, 21.1–25.3 for the chronic atrioventricular block canine heart, 17.2–25.4 for the normal human adult heart and 4.6–9.2 for the intact male New Zealand white rabbit heart. Thus, the termination of TdP may largely depend on the effective size of the heart for spiral rotation; namely, the smaller effective size will enhance the spontaneous termination of TdP.

### Limitation

First, the histological analysis and gene expression pattern obtained from two chronic atrioventricular block monkeys were not quantitative but qualitative observation because of the low sampling number due to 3R’s rule and lack of quantitative PCR. Second, further histological study in the chronic atrioventricular block hearts without TdP, and neurohumoral control data of intact animals might identify the additional conditions required to complete the pathological remodeling as a proarrhythmia model. Third, transmural mRNA expression level from endo-to epicardial myocardium should be analyzed to examine the proarrhythmic substrate of chronic atrioventricular block heart. Fourth, we qualitatively evaluated the gene expression changes, but did not quantitatively correlate them with QRS width/QT interval/QTcF changes. Fifth, we did not assess the time course of ANP during the initial 4 weeks after the onset of atrioventricular block, suggesting that we might have missed another peak of ANP.

## Conclusion

While previously described advantages of the monkey model were confirmed (Goto et al., 2020a; Goto et al., 2020b; Goto et al., 2021; Goto et al., 2022), we found that it took several months for the monkey heart to complete the pathological remodeling after the onset of atrioventricular block, that the success rate of the model creation was not so great as that of the canine model, and that reduction of ventricular repolarization reserve may play a pivotal role for the model to complete the remodeling process. Current findings would provide a fundamental knowledge for better utilizing the chronic atrioventricular block monkeys as a proarrhythmia model for detecting drug-induced TdP.

## Data Availability

The datasets presented in this study can be found in online repositories. The names of the repository/repositories and accession number(s) can be found below: https://www.ncbi.nlm.nih.gov/geo/, GSE199943.
