# Supplementary figures and images for: Characterization of pathological remodeling in the chronic atrioventricular block cynomolgus monkey heart
Source: Front Pharmacol. 2023 Jan 19;14:1055031. doi: 10.3389/fphar.2023.1055031 (PMC9892184; doi:10.3389/fphar.2023.1055031)

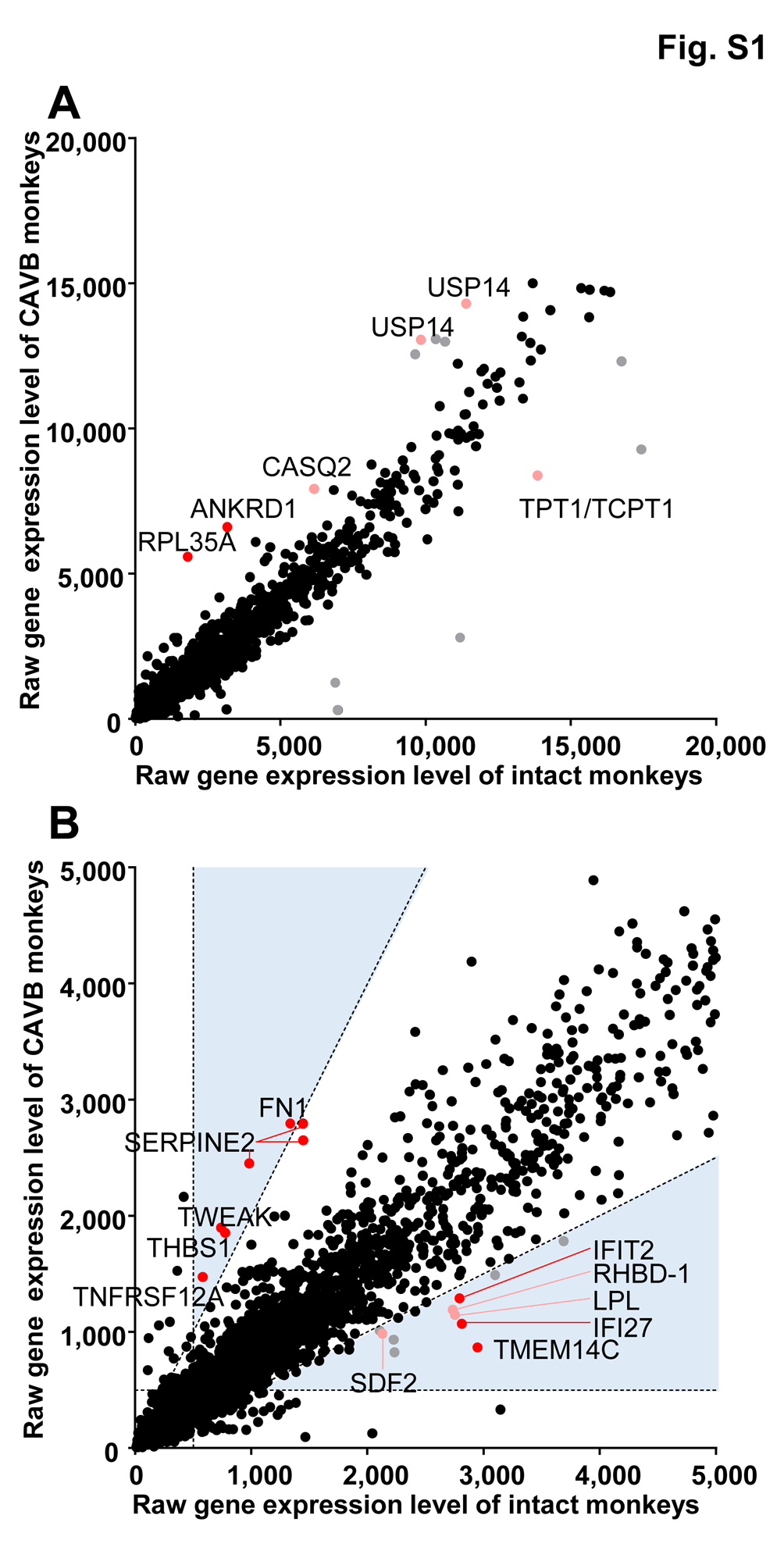

Supplement: Supplementary file 3 [file Image1.TIF]
