# Supplementary material for: Characterization of pathological remodeling in the chronic atrioventricular block cynomolgus monkey heart
Source: Front Pharmacol. 2023 Jan 19;14:1055031. doi: 10.3389/fphar.2023.1055031 (PMC9892184; doi:10.3389/fphar.2023.1055031)
Supplement: Supplementary file 5 [file DataSheet1.DOCX]

**Supplementary information**

**Table S1.** Durations of *dl*-solalol-induced torsade de pointes (TdP) observed in the animals #2, #3, #8 and #10.

**Table S2A.** Changes of hematological and biochemical factors in plasma

**Table S2B.** Changes of hematological and biochemical factors in plasma (*Continued*)

**Figure S1**. The comparison of averaged raw gene expression levels in the left ventricle between intact monkeys (n=4) and chronic atrioventricular block (CAVB) ones (n=2). (A) Information of the raw gene expression levels in the range of 5,000-20,000 was described. (B) Information of the raw gene expression levels in the range of 500-5,000 was depicted. The genes identified as functional proteins with ≥2 or ≤0.5-fold differences between the groups were shown in red (see Table 3); those identified as functional proteins with 1.5-2 or 0.5-0.7-fold differences between the groups were done in pink; and those not identified as functional proteins with ≥1.5 or ≤0.7 -fold differences between the groups were done in gray. Areas with light blue between the dotted lines indicate the raw gene expression levels of ≥500 in each group and raw gene expression ratio of CAVB monkeys to intact ones with ≥2 or ≤0.5.
